# pLG72 levels increase in early phase of Alzheimer’s disease but decrease in late phase

**DOI:** 10.1038/s41598-019-49522-1

**Published:** 2019-09-13

**Authors:** Chieh-Hsin Lin, Chih-Chiang Chiu, Chiung-Hsien Huang, Hui-Ting Yang, Hsien-Yuan Lane

**Affiliations:** 1 Department of Psychiatry, Kaohsiung Chang Gung Memorial Hospital, Chang Gung University College of Medicine, Kaohsiung, Taiwan; 2 Graduate Institute of Biomedical Sciences, China Medical University, Taichung, Taiwan; 3 School of Medicine, Chang Gung University, Taoyuan, Taiwan; 4 Department of Psychiatry, Taipei City Psychiatric Center, Taipei, Taiwan; 5 Department of Psychiatry, School of Medicine, Taipei Medical University, Taipei, Taiwan; 6 Department of Medicine Research, China Medical University Hospital, Taichung, Taiwan; 7 Department of Psychiatry, School of Medicine, Taipei Medical University, Taipei, Taiwan; 8 Department of Psychiatry & Brain Disease Research Center, China Medical University Hospital, Taichung, Taiwan; 9 Department of Psychology, College of Medical and Health Sciences, Asia University, Taichung, Taiwan

**Keywords:** Diagnostic markers, Alzheimer's disease

## Abstract

pLG72, named as D-amino acid oxidase activator (although it is not an activator of D-amino acid oxidase demonstrated by later studies), in mitochondria has been regarded as an important modulator of D-amino acid oxidase that can regulate the N-methyl-D-aspartate receptor (NMDAR). Both oxidative stress in mitochondria and NMDAR neurotransmission play essential roles in the process of neurodegenerative dementia. The aim of the study was to investigate whether pLG72 levels changed with the severity of neurodegenerative dementia. We enrolled 376 individuals as the overall cohort, consisting of five groups: healthy elderly, amnestic mild cognitive impairment [MCI], mild Alzheimer’s disease [AD], moderate AD, and severe AD. pLG72 levels in plasma were measured using Western blotting. The severity of cognitive deficit was principally evaluated by Clinical Dementia Rating Scale. A gender- and age- matched cohort was selected to elucidate the effects of gender and age. pLG72 levels increased in the MCI and mild AD groups when compared to the healthy group. However, pLG72 levels in the moderate and severe AD groups were lower than those in the mild AD group. D-serine level and D- to total serine ratio were significantly different among the five groups. L-serine levels were correlated with the pLG72 levels. The results in the gender- and age- matched cohort were similar to those of the overall cohort. The finding supports the hypothesis of NMDAR hypofunction in early-phase dementia and NMDAR hyperfunction in late-phase dementia. Further studies are warranted to test whether pLG72 could reflect the function of NMDAR.

## Introduction

Age-related cognitive decline is more and more prevalent in this rapidly aging world. Alzheimer’s disease (AD) is the most common cause of neurodegenerative dementia. Current diagnosis of mild cognitive impairment (MCI, a transitional state with minimal cognitive impairment and relatively intact daily functioning)^1^ or AD is mainly based on clinical assessment. Feasible laboratory tests in peripheral blood for assisting the diagnosis of MCI or AD will be beneficial for its early detection and intervention. Pathogenesis of neurodegenerative dementia is multi-factorial. In addition to the well-known amyloid-β hypothesis, dysregulation of glutamate neurotransmission is among the possible mechanisms^2^. Among the subtypes of ionotropic glutamate receptors, the N-methyl-D-aspartate receptor (NMDAR) plays an essential role in cognitive function, particularly memory and learning^3,4^. The energy metabolism may also be critical in the process of neurodegenerative dementia^5^.

Mitochondria are the main producers of energy and source of reactive oxygen species (ROS) in the cells^6^. pLG72, named as D-amino acid oxidase activator (although it is not an activator of D-amino acid oxidase demonstrated by later studies), is a protein that exists in mitochondria^7^ of four primate species including human being^8^. A post-mortem study showed that pLG72 protein expression in the brain was age dependent^9^. The *G72* gene was also reported to modify the age of onset in AD^10^ and affect the occurrence of psychotic symptoms in patients with AD^11^.

pLG72 has been proposed to interact with D-amino acid oxidase (DAAO)^12^. DAAO is capable of degrading D-amino acids including D-serine and D-alanine, which are co-agonists of the NMDAR^13^. DAAO concentration in peripheral blood has been found to reflect cognitive aging^4^. A DAAO inhibitor, sodium benzoate, showed beneficial effect for the cognitive and global function in patients with early phase dementia^14^. *G72* is a susceptibility gene for schizophrenia^7,15^. Sodium benzoate also showed efficacy for schizophrenia patients^16,17^. In fact, there are some similarities between schizophrenia and AD: both reveal cognitive and functional deficits^18–20^, behavioral problems^21^, implication with NMDAR^2,22^ and response to the DAAO inhibitor. Previous study found that pLG72 concentration in the peripheral blood was higher in patients with schizophrenia than in controls^23^. The aim of this study is to investigate whether pLG72 protein levels display a linear or nonlinear pattern in patients with neurodegenerative dementia.

## Results

Totally 376 participants were enrolled: 108 healthy elders (controls), 81 amnestic MCI patients, 124 mild AD patients, 35 moderate AD patients, and 28 severe AD patients.

### Unmatched cohort

There were more females in the controls than the other four AD groups (p = 0.015). The age distribution, education and MMSE scores were significantly different among the five groups (p < 0.001). The percentages of patients taking anti-dementia drugs (including memantine and AChEI) were different significantly among the four groups with cognitive deficits (p < 0.001). In the amino acids measured, the inter-groups differences were significant for D-serine level and D- to total serine ratio (p = 0.001, 0.018, respectively). The clinical and demographic characteristics are shown in Table 1.

**Table 1:** Demographic characteristics of the overall cohort (n = 376).

	Healthy elderly (CDR = 0, n = 108)	MCI (CDR = 0.5, n = 81)	Mild AD (CDR = 1, n = 124)	Moderate AD (CDR = 2, n = 35)	Severe AD (CDR = 3, n = 28)	*p* Value
Gender, female, n (%)	47 (43.5)	47 (58.0)	79 (63.7)	24 (68.6)	15 (53.6)	0.015^a^
Age, year, mean (SD)	67.2 (9.8)	68.2 (7.5)	73.6 (7.9)	79.5 (8.2)	77.8 (9.1)	<0.001^c^
Education, year, mean (SD)	11.2 (4.1)	6.7 (5.0)	5.0 (4.2)	5.1 (5.2)	5.8 (4.9)	<0.001^c^
MMSE, mean (SD)	28.1 (1.5)	23.3 (3.1)	18.9 (4.4)	11.5 (3.7)	8.1 (4.2)	<0.001^b^
**No. of subjects using anti-dementia drugs**						
Total number (%)	NA	9 (11.1)	43 (34.7)	4 (11.4)	6 (21.4)	<0.001^a*^
Donepezil (dose, mean ± SD)	NA	8 (6.9 ± 2.6)	28 (9.1 ± 2.0)	1 (10.0 ± 0.0)	4 (10.0 ± 0.0)	0.011^a*^
Rivastigmine (dose, mean ± SD)	NA	1 (9.0)	6 (6.8 ± 2.5)	0	2 (5.5 ± 0.7)	0.220^a*^
Galantamine (dose, mean ± SD)	NA	0	9 (15.1 ± 2.7)	1 (16.0)	0	0.035^a*^
Memantine (dose, mean ± SD)	NA	0	0	2 (20.0 ± 0.0)	0	0.004^a*^
pLG72 level (ng/mL), mean (SD)	1.4 (0.7)	2.3 (1.1)	2.9 (1.6)	2.7 (1.4)	2.0 (1.3)	<0.001^c^
pLG72 with anti-dementia drugs	NA	2.3 (1.1)	2.7 (1.9)	2.3 (1.0)	2.7 (1.8)	0.999^c*^
pLG72 without anti-dementia drugs	1.4 (0.7)	2.3 (1.1)	3.0 (1.4)	2.7 (1.4)	1.8 (1.1)	0.001^b^
Glycine level (ng/mL), mean (SD)	3815.9 (1333.0)	4198.5 (1351.1)	4892.4 (2091.9)	4949.4 (2252.8)	4214.2 (1120.5)	0.061^b^
L-serine level (ng/mL), mean (SD)	2858.3 (790.7)	3601.8 (1537.5)	3579.2 (1197.2)	3303.9 (1211.9)	3267.6 (1368.3)	0.087^b^
D-serine level (ng/mL), mean (SD)	30.8 (11.6)	44.6 (25.8)	49.2 (27.2)	54.0 (26.9)	60.8 (25.8)	0.001^b^
L-alanine level (ng/mL), mean (SD)	11347.0 (3250.9)	11643.3 (3043.1)	12532.0 (3424.6)	12221.2 (3242.2)	12764.5 (3493.5)	0.476^b^
D-alanine level (ng/mL), mean (SD)	30.2 (38.0)	35.1 (34.2)	31.0 (36.0)	45.4 (33.8)	39.0 (49.4)	0.602^b^
D/T-serine ratio, mean (SD)	0.011 (0.005)	0.014 (0.009)	0.014 (0.008)	0.017 (0.009)	0.020 (0.009)	0.018^b^
D/T-alanine ratio, mean (SD)	0.003 (0.003)	0.003 (0.004)	0.003 (0.003)	0.004 (0.003)	0.003 (0.004)	0.899^b^

NA, not associated; ^a^Chi-square test; ^b^ANOVA test; ^c^Kruskal-Wallis test; ^*^Comparison among MCI, mild, moderate and severe AD groups.

Abbreviations: CDR, Clinical Dementia Rating; MMSE, Mini Mental Status Examination; pLG72, D-amino acid oxidase activator; T-serine, total serine; T-alanine, total alanine; D/T-serine ratio, D-serine/total serine ratio; D/T-alanine ratio, D-alanine/total alanine ratio.

### Matched cohort

The five groups were matched further for age and gender. The gender and age distributions were not significantly different among the five groups in the matched cohort (p = 0.126, 0.109, respectively). The education level of the controls was significantly higher than the four groups with cognitive deficits (p < 0.001). The percentages of patients taking anti-dementia drugs were significantly different among the four groups with cognitive deficits (p = 0.018). For the five amino acids in the matched cohort, the inter-groups difference was significant for D-serine level (p = 0.015). The clinical and demographic characteristics are shown in Table 2 (matched cohort).

**Table 2:** Demographic characteristics of the gender- and age- matched cohort (n = 242).

	Healthy elderly (CDR = 0, n = 56)	MCI (CDR = 0.5, n = 45)	Mild AD (CDR = 1, n = 99)	Moderate AD (CDR = 2, n = 20)	Severe AD (CDR = 3, n = 22)	*p* Value
Gender, female, n (%)	23 (41.1)	28 (62.2)	61 (61.6)	12 (60.0)	12 (54.5)	0.126^a^
Age, year, mean (SD)	72.9 (9.9)	72.2 (6.6)	73.2 (7.1)	77.2 (4.8)	75.4 (7.2)	0.109^c^
Education, year, mean (SD)	9.6 (4.2)	5.4 (3.9)	5.6 (4.5)	4.9 (5.2)	5.2 (5.1)	<0.001^c^
MMSE, mean (SD)	27.6 (1.6)	23.1 (3.2)	19.3 (4.2)	11.9 (4.4)	7.6 (4.3)	<0.001^b^
**No. of subjects using anti-dementia drugs**						
Total number (%)	NA	6 (13.3)	34 (34.3)	2 (10.0)	6 (27.3)	0.018^a*^
Donepezil (dose, mean ± SD)	NA	6 (6.7 ± 2.6)	19 (8.9 ± 2.1)	0	4 (10.0 ± 0.0)	0.175^a*^
Rivastigmine (dose, mean ± SD)	NA	0	6 (6.8 ± 2.5)	0	2 (5.5 ± 0.7)	0.180^a*^
Galantamine (dose, mean ± SD)	NA	0	9 (15.1 ± 2.7)	1 (16.0)	0	0.090^a*^
Memantine (dose, mean ± SD)	NA	0	0	1 (20.0)	0	0.039^a*^
pLG72 level (ng/mL), mean (SD)	1.4 (0.6)	2.3 (1.0)	2.9 (1.5)	2.4 (1.3)	2.1 (1.3)	<0.001^c^
pLG72 with anti-dementia drugs	NA	2.2 (1.3)	2.7 (1.6)	1.9 (1.2)	2.7 (1.8)	0.875^c*^
pLG72 without anti-dementia drugs	1.4 (0.6)	2.3 (1.0)	3.0 (1.4)	2.5 (1.3)	1.9 (1.1)	<0.001^b^
Glycine level (ng/mL), mean (SD)	3687.6 (1009.2)	4125.3 (1316.5)	4973.3 (2171.8)	4748.6 (1307.8)	4254.4 (879.8)	0.137^b^
L-serine level (ng/mL), mean (SD)	2812.9 (928.3)	3471.5 (1598.9)	3563.4 (1102.5)	3427.4 (1201.6)	3440.5 (1410.4)	0.407^b^
D-serine level (ng/mL), mean (SD)	31.2 (9.5)	38.3 (15.6)	50.0 (27.7)	59.9 (30.9)	59.5 (25.6)	0.015^b^
L-alanine level (ng/mL), mean (SD)	11324.6 (3337.0)	11704.3 (2809.7)	12524.7 (3180.9)	11575.1 (3176.4)	13064.4 (3591.8)	0.558^b^
D-alanine level (ng/mL), mean (SD)	26.6 (38.4)	26.3 (23.2)	28.9 (31.2)	52.1 (42.0)	28.1 (22.2)	0.234^b^
D/T-serine ratio, mean (SD)	0.012 (0.005)	0.013 (0.007)	0.014 (0.008)	0.018 (0.010)	0.018 (0.009)	0.182^b^
D/T-alanine ratio, mean (SD)	0.002 (0.003)	0.002 (0.002)	0.003 (0.003)	0.004 (0.003)	0.002 (0.002)	0.307^b^

NA, not associated; ^a^Chi-square test; ^b^ANOVA test; ^c^Mann-Whitney U test; ^*^Comparison among MCI, mild, moderate and severe AD groups.

Abbreviations: CDR, Clinical Dementia Rating; MMSE, Mini Mental Status Examination; pLG72, D-amino acid oxidase activator; T-serine, total serine; T-alanine, total alanine; D/T-serine ratio, D-serine/total serine ratio; D/T-alanine ratio, D-alanine/total alanine ratio.

### pLG72 levels were highest in mild AD patients

**Figure 1:**
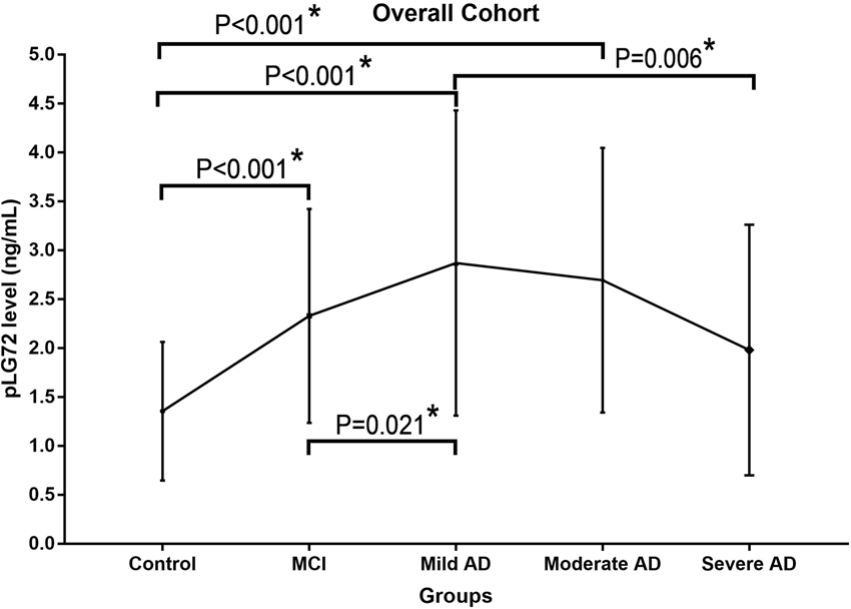
MCI, mild cognitive impairment; AD, Alzheimer’s disease. The error bars represent standard deviation. *P < 0.05.

The pLG72 levels of the healthy elders, amnestic MCI, mild AD, moderate AD, and severe AD were 1.4 ± 0.7 ng/mL, 2.3 ± 1.1 ng/mL, 2.9 ± 1.6 ng/mL, 2.7 ± 1.4 ng/mL and 2.0 ± 1.3 ng/mL, respectively (p < 0.001) (Table 1 and Fig. 1). Bonferroni method was used for post-hoc analysis. The result revealed that the pLG72 levels in control group were lower than those in amnestic MCI, mild AD, and moderate AD (p < 0.001, <0.001, <0.001, respectively). The pLG72 levels in mild AD were significantly higher than those in amnestic MCI and in severe AD (p = 0.021, 0.006, respectively). The inter-groups differences of pLG72 levels among other groups were not significant (p > 0.05). The pLG72 levels in participants with or without anti-dementia agents were not significantly different (Table 1).

**Figure 2:**
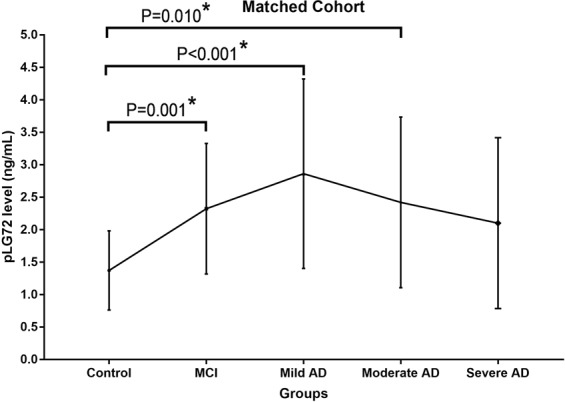
MCI, mild cognitive impairment; AD, Alzheimer’s disease. The error bars represent standard deviation. *P < 0.05.

In the gender- and age- matched cohort, the pLG72 levels in the controls, amnestic MCI, mild AD, moderate AD and severe AD were 1.4 ± 0.6 ng/mL, 2.3 ± 1.0 ng/mL, 2.9 ± 1.5 ng/mL, 2.4 ± 1.3 ng/mL and 2.1 ± 1.3 ng/mL, respectively (p < 0.001) (Table 2 and Fig. 2). Post-hoc analysis (Bonferroni method) revealed that the pLG72 levels in controls were lower than those in amnestic MCI, mild AD and moderate AD (p = 0.001, <0.001, 0.010, respectively). The inter-groups differences of pLG72 levels among other groups were not significant (p > 0.05). The pLG72 levels in participants with and without anti-dementia agents were also not significantly different in the matched cohort (Table 2).

### The relationship between pLG72 level and L-serine levels

Multiple linear regression analyses were used to test the relationship between pLG72 and amino acids. Age and sex were adjusted in the regression models for the overall cohort. Because the co-linearity was high between D- to L-form ratios and amino acids levels, amino acids levels only but not ratios were included in the models. There were significant associations between pLG72 levels and age and L-serine levels in the overall cohort (adjusted R^2^ = 0.114) (Table 3). For the matched cohort, age and sex were not adjusted in the regression models. There was also significant association between pLG72 levels and L-serine levels in the matched cohort (adjusted R^2^ = 0.035) (Table 3).

**Table 3:** Multiple linear regression analyses of independent factors associated with pLG72 level in overall and matched cohorts (stepwise).

Variable	B (SE)	t	*P*
**Overall cohort (N = 376)**			
Age	0.033 (0.010)	3.312	0.001
L-serine level (ng/mL)	0.000 (0.000)	2.672	0.008
Adjusted R square = 0.114			
**Matched cohort (N = 242)**			
Variable	B (SE)	t	*P*
L-serine level (ng/mL)	0.000 (0.000)	2.136	0.035
Adjusted R square = 0.035			

The regression model was adjusted with age and sex for the overall cohort. The variables were L-serine level, D-serine level, glycine level, L-alanine level, D-alanine level, D/T-serine ratio and D/T-alanine ratio. Significant variables are shown in the Table (p < 0.05).

## Discussion

The results of this study in the elderly population showed that pLG72 protein levels reveal a non-linear association with the severity of cognitive decline. In healthy elders and patients with early-phase dementia (including MCI and mild AD), the pLG72 levels increased when the cognitive deficits were worsened, with the highest level in mild AD. However, the pLG72 levels decreased in patients with moderate or severe AD when compared to those in early-phase dementia. There was a weak association between pLG72 levels and L-serine levels in peripheral blood. Furthermore, the results were similar in both the unmatched cohort and the age- and gender-matched cohort. This novel finding suggests that pLG72 may play a role in the process of neurodegenerative dementia possibly related to the NMDAR modulation.

The function of pLG72 protein in brain and its disorders is still an enigma^24^. pLG72 protein is detectable in many brain regions such as cerebellum, striatum and frontal cortex^9^. However, it is not yet clear which cells in the brain contain pLG72 protein. Further study is warranted to find out specific cells containing pLG72 protein. Considering its exclusive existence in human being and three other primates, pLG72 protein may be important for advanced cognitive functions. pLG72 plays a pivotal role in the modulation of NMDAR via DAAO activation^25^. Accumulating evidence suggests that the function of NMDAR decreases in patients with cognitive decline. The density of NMDARs decreases during the normal process of aging^26^. In patients with AD, the number of glutamate terminals reduces in the hippocampus^27^. While some study found that the levels of D-serine (the primary co-agonist of NMDAR) slightly fell and L-serine slightly rose in the serum^28^, other studies noted that D-serine levels increased in the brain, CSF^29^ and serum in AD patients^4^. The peripheral DAAO levels were found to be higher in the patients with MCI or AD than the healthy elderly individuals^4^. “Glutamate excitotoxicity theory” is one of the possible pathogeneses of AD, particularly in the later phase. Memantine, a partial NMDAR antagonist that blocks NMDAR overactivation^30–32^, has been approved for use as a medication for moderate-severe AD^33^. We hypothesize that pLG72 may play a pivotal role on the modulation of NMDAR function in neurodegenerative dementia, yet its function in different phases of dementia has not yet been clear. Further studies are warranted for verification.

In addition to its role in modulating DAAO, pLG72 is also a mitochondrial protein that regulates the production of reactive oxygen species^34^. A cell line study using systemic approaches indicated that pLG72 might be involved in the induction of ROS^35^. Another study found that pLG72 interacted with a mitochondrial protein (methionine-R-sulfoxide reductase B2) that functioned in oxidative stress defense^36^. Oxidative stress has been regarded to contribute to aging with the assumption that free radicals damage cell constituents and connective tissues^37^. Increased oxidative stress might play an important role underlying processes of aging or neurodegenerative diseases^38,39^. Age-related cognitive decline is correlated with a decrease in brain and plasma antioxidants, for example, glutathione (GSH)^40–42^. The role of pLG72 in the regulation of oxidative stress that occurs in the process of neurodegenerative dementia deserves further elucidation.

D-serine level and D- to total serine ratio were significantly different among the five groups of participants. Results from the present study revealed a significant but weak association between pLG72 level and L-serine level (the adjusted R squares was low) but not D-serine (Table 3). The relationship between pLG72 and L-serine needs to be confirmed in further studies with larger sample sizes. Previous study showed that patients with congenital defects in the L-serine synthesizing enzymes manifest severe neurological abnormalities^43^. The role of L-serine in neurodegenerative dementia has not yet been clear. D-serine is a major endogenous co-agonist of the NMDAR. pLG72 has been thought to be able to modulate the D-serine metabolism^44^. Of note, D-serine is also regulated by many other mechanisms such as serine racemase that synthesizes D-serine from L-serine^45^. Serine racemase has also been thought to play a role in the aging process^46^. Other modulatory mechanisms should be also considered when the relationship between pLG72 and D-serine and L-serine is explored. In addition, the relationship between pLG72 level and severity of cognitive deficits was non-linear in this study. The non-linear dynamics has been observed in many research fields including neuroscience and cognitive psychology^47^. The non-linearity phenomenon may reflect the dynamic complexity of brain fine-tuning function^48^. Analyses that are appropriate for approaching biological non-linearity will be helpful in exploring the interactions among pLG72 and amino acids in future longitudinal studies^49^.

This study has several limitations. Firstly, the finding from this study is limited by its cross-sectional design. Secondly, the peripheral blood-CNS relationship of pLG72 requires investigation in patients with neurodegenerative dementia. Thirdly, only Han Chinese populations were recruited in this study. The findings need to be tested in other populations. Fourthly, the sample sizes in the moderate AD and the severe AD groups were relatively small that might hinder us to draw definite conclusion. Fifthly, the education levels between the control group and the cognitively impaired group were different. Although the grouping of participants was based on CDR score which is not influenced by education^50^, further study on participants with matched education levels is warranted for further elucidating the education effect on pLG72 levels. Lastly, although we had performed a thorough physical and mental work-up to exclude patients with comorbid mental or organic disorders, it was not possible to exclude with certainty all other neurodegenerative diseases (e.g. amyotrophic lateral sclerosis) that may alter pLG72 levels^51^.

In summary, this study suggests that the pLG72 levels in the peripheral blood increase in patients with early-phase dementia with the peak at mild AD, but decrease with the severity of cognitive deficits in later phase of AD. The findings which need to be confirmed and explored for underlying mechanisms by further studies indirectly supports the hypo-NMDAR hypothesis in early-phase AD and glutamate excitotoxicity hypothesis in late-phase AD. In the future, combining pLG72 level with other potential biomarkers such as DAAO and β-amyloid^52^ levels for assisting the diagnosis of AD particularly in its early phase may be favorable. For example, molecules that bind the DAAO-pLG72 complex have been discovered^53^. The fluctuation of pLG72 concentration in the progression from the early phase of AD to its later phase needs to be confirmed in prospective longitudinal, larger-scale studies. It is worthy to test whether pLG72 could serve as a biomarker reflecting the function of NMDAR. Finally, it is also interesting to explore whether pLG72 blockers can be developed as a treatment for AD in its early phase and whether pLG72 enhancers, as a treatment for AD in its late phase^54,55^.

## Materials and Methods

### Participants

All participants were recruited and evaluated from Kaohsiung Chang Gung Memorial Hospital, Kaohsiung, Taiwan, China Medical University Hospital, Taichung, Taiwan, and Taipei City Municipal Hospital, Taipei, Taiwan. This study was carried out in accordance with the recommendations of Good Clinical Practice (GCP), Institutional Review Board of Kaohsiung Chang Gung Memorial Hospital, Institutional Review Board of China Medical University Hospital, and Institutional Review Board of Taipei City Municipal Hospital, Taiwan. The protocol was officially approved by the institutional review boards of these hospitals. All subjects provided written informed consent according to the Declaration of Helsinki.

All participants were 50–100-year-old Han Chinese who were physically healthy with normal blood routine and biochemical tests. All participants were evaluated by research physicians thoroughly. Participants were recruited if they [1] had adequate education for effective communication, [2] were able to complete the evaluations in this study, and [3] provided written informed consent to join this study. The exclusion criteria included major medical, neurological, or psychiatric disorders other than AD; delirium symptoms; substance dependence or abuse (including alcohol); Hachinski Ischemic Score >4; history of significant cerebrovascular disease; severe hearing or visual impairment; and being unable to follow protocol. The healthy volunteers were free from any psychiatric disorder. Similarly, all healthy participants had no substance abuse or dependence (including alcohol) diagnosed by DSM-IV.

- Patients with MCI satisfied amnestic MCI criteria^56^, in which a probable degeneration course including subjective memory decline with a Clinical Dementia Rating (CDR)^57^ score 0.5 is noted, but the impairments in cognitive and global function are insufficient to meet the NINCDS-ADRDA criteria (National Institute of Neurological and Communicative Disorders and Stroke and the Alzheimer’s Disease and Related Disorders Association)^20^.
- Patients with mild AD satisfied NINCDS-ADRDA criteria for probable AD with a CDR score 1.
- Patients with moderate AD satisfied NINCDS-ADRDA criteria for probable AD with a CDR score 2.
- Patients with severe AD satisfied NINCDS-ADRDA criteria for probable AD with a CDR score 3.
- Healthy individuals had a CDR score 0.

All patients with AD were enrolled at the departments of outpatient clinic of the aforementioned hospitals. The healthy individuals were enrolled in the communities of southern, central and northern Taiwan.

AD patients with and without anti-dementia drugs were both recruited. AD patients without anti-dementia drugs were free from those medications for three months or longer. For patients with anti-dementia drugs treatment, those medications had been maintained for three months or longer with unchanged doses. Medication history was determined by reviewing medical records, confirming with providers of health care, and history taking with the participants and their caregivers or family. Healthy individuals were free from anti-dementia drugs.

### The assessments of cognitive function

The cognitive functions of the participants were evaluated by Mini-Mental State Examination (MMSE)^58^ and CDR. MMSE is common for screening dementia and measuring cognition^58^. Nevertheless, MMSE is easily influenced by education and age^59^, and has low sensitivity for mild cognitive deficits, therefore limiting its use^60^.

In contrast, CDR has good discrimination power for dementia with slight impairment^61^. Further, CDR has shown good reliability and validity for the assessment and staging of dementia with favorable inter-rater reliability^62^. Thus, the grouping of participants was mainly based upon CDR that represented both the cognitive and global impairment of the participants. Of note, CDR score is not influenced by age, gender and education^50^.

### Laboratory assessments

#### pLG72 protein level measurement

Well-trained personnel collected subjects’ peripheral blood (10 ml) into EDTA-containing blood collection tubes. The specimens were immediately centrifuged at 500 g. Plasma was dissected quickly and stored immediately at −80 °C after centrifugation until Western blotting.

The plasma pLG72 protein levels were examined by Western blotting^23^. At first, 100 µl plasma was depleted using ProteoPrep® Blue Albumin and IgG Depletion Kit (Sigma). The low-abundant protein fractions were collected to 100 μl. Then, 10 μl of the fractions were mixed with 4X sample buffer (500 mM Tris-HCl (pH 6.8), 16% SDS, 80% glycerol, 400 mM DTT, and 0.08% bromophenol blue) and separated on 12% SDS-PAGE. Proteins in the gels were transferred to 0.45 μm polyvinylidene difluoride (PVDF) membrane (Millipore), which was placed in 5% nonfat dry milk in TBST (20 mM Tris-HCl pH 7.6, 500 mM sodium chloride, 0.1% Tween 20) for 1 hour at room temperature, then incubated with goat anti-pLG72 antibody (pLG72 (N15):sc-46118, Santa Cruz Biotechnology) diluted by 1:1000 in TBST overnight at 4 °C. The membrane was washed thrice in TBST and incubated for 2 hours with an HRP-linked anti-goat IgG secondary antibody (sc-2030, Santa Cruz Biotechnology) diluted by 1:5000 in TBST. After 3 washes in TBST, the blots were visualized with an ECL Advance Western Blotting Detection Kit (RPN2135, GE Healthcare). The stained membranes were photographed on ImageQuant LAS 4000 mini (GE Healthcare) and quantified using ImageQuant™ TL 7.0 software (GE Healthcare) by measuring the relative intensity from each band and normalized to the pLG72 recombinant protein (20 ng) signals. The commercial pLG72 antibodies were able to specifically recognize LG72 recombinant proteins. A standard curve was generated by serial dilutions of the pLG72 protein (50, 20, 10, 5, 2.5, 1.25, and 0.625 ng), and its detection limit was as low as 0.625 ng (Supplementary Fig. 1). The Western blotting was repeated by two experienced technicians separately for quality control. The results of the Western blotting were very similar between the two technicians. The R^2^ of the linearity between the Western blotting signals and the amounts of the pLG72 proteins was 0.988. In the Western blotting, the molecular weight of the pLG72 protein band was approximately 18 kDa. The molecular weight of the standard recombinant pLG72 protein (as control) which had a tagged protein on it was marginally higher than that of the plasma pLG72 protein (Supplementary Fig. 2). The noise-signal ratios around the points of the Western blotting were between 0.04 and 0.13. All Western blot analyses were repeated twice.

#### Amino acids levels measurement

Serum was firstly extracted by methanol (1:3, by volume), then filtered after 15 min centrifugation (1500 × g) with nylon membranes (0.45 mM, Minisart SRP4, Sartorius, Germany). The filtrate was diluted with proper amount of 20% methanol then derivatized with N-isobutyl-L-cysteine (IBC) and O-phthaldialdehyde (OPA) mixture for 5 minutes then injected into high performance liquid chromatography (HPLC, L-7100 Pump, L7250 Autosampler, L-7250, with L7480 fluorescence Detector, Hitachi, Japan) for analysis. Analytical column (Grom-Sil OPA-2, 5 µm, 250 mm * 4 mm, Part No: GSOP 20512S2504, SAP No: 5113679, Grace, US) with guard column (Grom-Sil OPA-2, 5 µm, 10 mm × 4 mm, Part No: GSOP20512v0104V, Grace, US) were used for the determination. Isocratic elution of mobile phase A (23 mM sodium acetate, pH 6.0) and B (50 mL acetonitrile in 600 mL methanol) were performed under fluorescence detection (excitation 260 nm, emission 455 nm), respectively. Retention time of each amino acid was L-serine, 33.6 min; D-serine, 35.8 min; glycine, 41.5 min; L-alanine, 47.2 min; D-alanine, 50.3 min, respectively. All amino acids levels were double-checked by performing HPLC analyses for two times in order to confirm that the peaks were not artifact (Supplementary Fig. 3).

We also used 10 µL D-amino acid oxidase (DAAO) mixed with 40 µL serum sample for the verification of D-amino acids levels^63^. After serial gradient heating, 150 mL methanol was added and centrifuged under 15000 × g at 4°C for 15 minutes. The filtrate was injected into the HPLC when reacting with OPA. D-serine and D-alanine levels were markedly decreased after DAAO addition. The levels of L-form amino acids and D-glutamate were slightly decreased, representing the diluting effect in the test (Supplementary Table 1).

### Statistical analysis

All participants’ demographic, clinical characteristics and laboratory measures are shown as number (percentage) or mean ± SD. All percentages between groups were compared by χ2 test, and mean values among groups were compared by one-way ANOVA. For three or more groups, Kruskal-Wallis test was used. Multiple linear regression analysis was used to generate correlation models for pLG72 levels and amino acids levels. Statistical significance was defined as a p value ≤ 0.05. IBM SPSS Statistics (version 22.0, SPSS inc.) was applied to conduct all statistical analyses.

### Role of the sponsor

The sponsors were not involved in the design and conduct of the study; collection, management, analysis, and interpretation of the data; and preparation, review, or approval of the manuscript.

## Supplementary information

Supplementary information
