# Supplementary material for: pLG72 levels increase in early phase of Alzheimer’s disease but decrease in late phase
Source: Sci Rep. 2019 Sep 13;9:13221. doi: 10.1038/s41598-019-49522-1 (PMC6744481; doi:10.1038/s41598-019-49522-1)
Supplement: Supplementary file 1 — Supplementary information [file 41598_2019_49522_MOESM1_ESM.docx]

**pLG72 levels increase in early phase of Alzheimer's disease but decrease in late phase**

Chieh-Hsin Lin, M.D., Ph.D. ^a, b, c^, Chih-Chiang Chiu, M.D., Ph.D. ^d, e^, Chiung-Hsien Huang ^f^, Hui-Ting Yang, Ph.D. ^g^, Hsien-Yuan Lane, M.D., Ph.D. ^b, h, i^*

^a^ Department of Psychiatry, Kaohsiung Chang Gung Memorial Hospital, Chang Gung University College of Medicine, Kaohsiung, Taiwan

^b^ Graduate Institute of Biomedical Sciences, China Medical University, Taichung, Taiwan

^c^ School of Medicine, Chang Gung University, Taoyuan, Taiwan

^d^ Department of Psychiatry, Taipei City Psychiatric Center, Taipei, Taiwan

^e^ Department of Psychiatry, School of Medicine, Taipei Medical University, Taipei, Taiwan

^f^ Department of Medicine Research, China Medical University Hospital, Taichung, Taiwan

^g^ Department of Psychiatry, School of Medicine, Taipei Medical University, Taipei, Taiwan

^h^ Department of Psychiatry & Brain Disease Research Center, China Medical University Hospital, Taichung, Taiwan

^i^ Department of Psychology, College of Medical and Health Sciences, Asia University, Taichung, Taiwan.

Running Title: pLG72 differs between early and late AD

* Corresponding author at: Department of Psychiatry, China Medical University Hospital, No. 2, Yuh-Der Road, Taichung 404, Taiwan. *Email address:* hylane@gmail.com (H.-Y. Lane).

**Supplementary Figure 1**. A standard curve was generated by serial dilutions of the pLG72 recombinant protein. The spots indicate different concentrations of pLG72 recombinant protein (50, 20, 10, 5, 2.5, 1.25, and 0.625 ng, respectively). The Western blotting showed that the commercial pLG72 antibodies were able to specifically recognize serially diluted pLG72 recombinant proteins and its detection limit was as low as 0.625 ng.


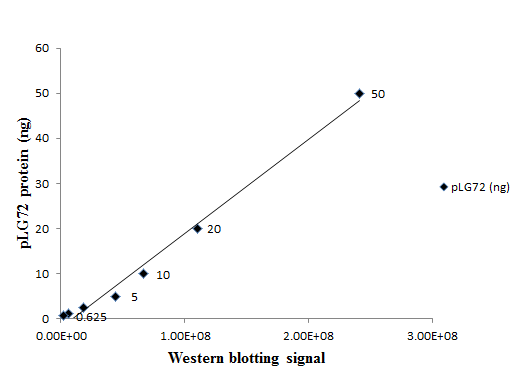


pLG72

1 2 3 4 5 6


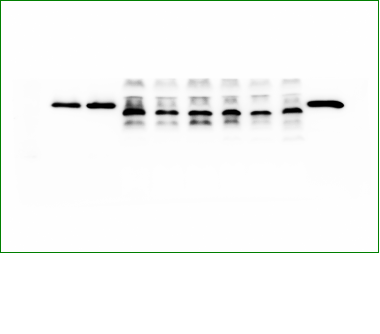


20ng

**Supplementary Figure 2**. Western blotting results of pLG72 protein in plasma from patients with neurodegenerative dementia (n=6).


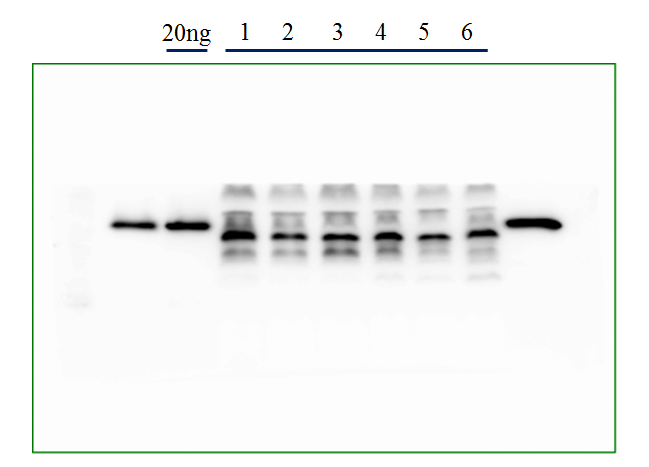


**Supplementary Figure 3**. Serum D-form and L-form amino acid profiles shown in HPLC spectrum. Red-lined spectrum represented D-, L- amino acid profiles of serum sample. Blue-lined spectrum represented D-, L- amino acid profiles with DAAO addition to serum sample.


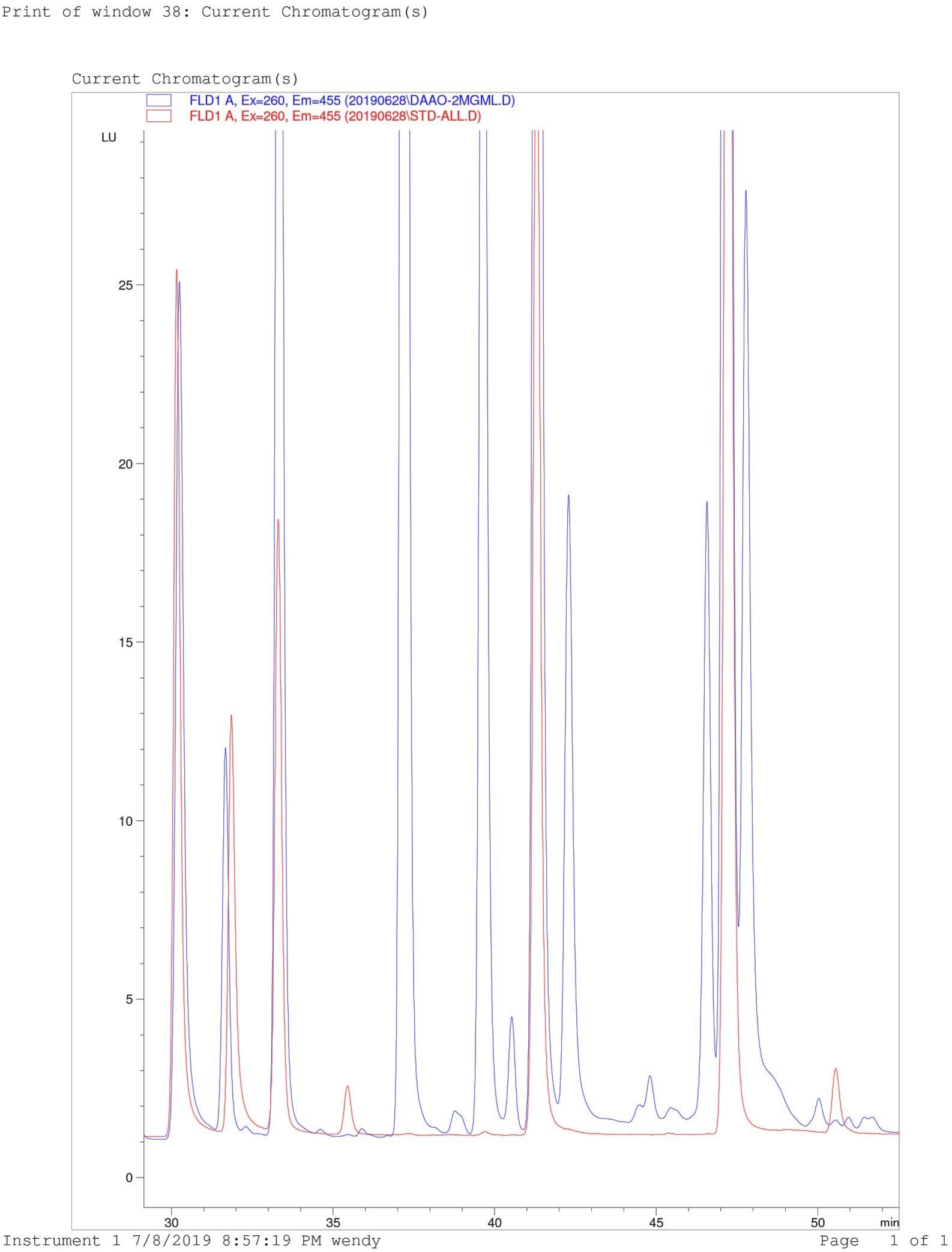


L-Glu

D-Glu

L-Ser

D-Ser

Gly

L-Ala

D-Ala

**Supplementary Table 1**. Area under curve (AUC) of each amino acid peak shown in the HPLC spectrum.

|  | L-Glutamate | D-Glutamate | L-Serine | D-Serine | Glycine | L-Alanine | D-Alanine |
| --- | --- | --- | --- | --- | --- | --- | --- |
| Without adding DAO† (PBS* only) | 445.6 | 156.0 | 893.4 | 10.4 | 1730.7 | 2153.9 | 9.0 |
| Adding DAO‡ | 428.4 | 150.7 | 871.5 | 0.6 | 1594.4 | 2077.9 | 3.5 |
| Decreased % of AUC | 3.9 | 3.4 | 2.5 | 94.2 | 7.9 | 3.5 | 61.1 |

† 40 µL serum were mixed with 10 µL of PBS.

‡ 40 µL serum were mixed with 10 µL of DAO (2 mg/mL).

* PBS, phosphate buffer saline.
